# Comprehensive analysis of dynamic gene expression and investigation of the roles of hydrogen peroxide during adventitious rooting in poplar

**DOI:** 10.1186/s12870-019-1700-7

**Published:** 2019-03-12

**Authors:** Yan Zhang, Zheng’ang Xiao, Chang Zhan, Meifeng Liu, Wenxiu Xia, Nian Wang

**Affiliations:** 1 College of Horticulture and Forestry Sciences, Huazhong Agricultural University, Wuhan, 430070 China; 2 Hubei Engineering Technology Research Center for Forestry Information, Huazhong Agricultural University, Wuhan, 430070 China

**Keywords:** Poplar, Adventitious rooting, Gene expression, Phytohormone, Hydrogen peroxide

## Abstract

**Background:**

Adventitious roots (ARs) are roots that are generated from nonrooting tissues. ARs are usually produced both during normal development and in response to stress conditions, such as flooding, nutrient deprivation, heavy metal stress and wounding. The ability of plants to form ARs is a key trait that enables plant propagation, especially for most tree species.

**Results:**

Here, the kinetics of AR formation in a tissue culture of a hybrid variety of poplar were investigated. AR formation mainly occurred during the first 8 days and both pre- and newly- formed primordia contributed to AR formation in poplar by histological study. RNA-Seq-based transcriptome analysis was performed for stem bases collected at 0, 2, 4, 6 and 8 days after excision (DAE). Based on the data, the expression patterns of 8 phytohormone-related genes were investigated, and their influences on AR formation were considered. Subsequent gene expression cluster analysis showed a number of biological processes involved in AR formation. Among these biological pathways, genes involved in H_2_O_2_ homeostasis showed enrichment in one cluster that was highly upregulated from DAE0 to DAE8. Pharmacological assay confirmed that an appropriate content of H_2_O_2_ in stem bases could accelerate the formation of ARs in poplar.

**Conclusions:**

Based on the results of this study, we were able to predict a regulatory network for 7 phytohormones that are involved in poplar AR formation. The influence of H_2_O_2_ on AR formation was also confirmed_._ These results enhance our understanding of the regulation of AR formation in tree species.

**Electronic supplementary material:**

The online version of this article (10.1186/s12870-019-1700-7) contains supplementary material, which is available to authorized users.

## Background

Adventitious roots (ARs) are roots that are generated both in vitro and in field cultivation from nonrooting tissues such as stems, leaves and hypocotyls. ARs are usually produced both during normal development and in response to stress conditions, such as flooding, nutrient deprivation, heavy metal stress and wounding [1]. The formation of ARs is one of the most important biological processes in most trees and some herbaceous plants, which are vegetative propagated by semi-hardwood cuttings or by microcuttings if under an in vitro system. Therefore, in some plants, the ability to form ARs is the key trait enabling their propagation. During the past several decades, many studies have focused on determining the mechanisms involved in AR formation in herbaceous plants such as *Arabidopsis thaliana*, *Oryza sativa* and *Petunia* hybrids [2, 3]. Generally, three factors are attributed to controlling AR formation, including genetic, environmental and physiological factors (e.g., phytohormones, phenolic compounds and nitrogen oxide) [2, 4]. All 3 of these factors and their components interact with each other and form a complex network that regulates AR formation. However, the understanding of AR formation in tree species is relatively limited. Though the details of this biological process may be shared between herbaceous and woody plants to some extent, there are considerable differences between these two types of plants; for example, the ability of trees to form ARs usually declines with age; while there is absent of juvenility for most herbaceous plants [5–7]. Thus, an investigation of the mechanisms involved in AR formation in trees is necessary.

Phytohormones, also known as plant hormones, play important roles in plant growth and environmental responses. Phytohormones act as the most important endogenous factors controlling the process of AR formation in plants. Among the diverse phytohormones, auxin has been confirmed to play a central role in AR formation in many studies [8]. Generally, the tissues that produce ARs require high auxin levels, and this high content of auxin is usually dependent on polar auxin transport (PAT) [9, 10]. Interestingly, excessively high auxin levels have been observed to have an inhibitory role at later stages of AR formation [11]. Cytokinins, which compose another key phytohormone group, regulate the division and differentiation of plant cells. In the early stages of AR induction in carnation, accumulation of cytokinins increases, while the accumulation decreases during the middle stages [12]. In addition to these two phytohormones, abscisic acid (ABA), brassinosteroids (BRs), ethylene (ET), gibberellins (GAs), jasmonic acid (JA) and salicylic acid (SA) have also been found to play roles during AR formation. Generally, a large and complex network is formed among these phytohormones, and this network complicates the phytohormones’ influence on AR formation. For example, interactions between ET, GAs and ABA regulate the emergence and growth rate of ARs in deepwater rice [13]. GAs inhibit adventitious rooting in poplar and Arabidopsis by affecting auxin transport [14]. In petunia, JA acts as a positive regulator of AR formation in cuttings [15]. Since there are interactions among all these phytohormones, BRs and SA may also be involved in the regulation of AR formation. However, the understanding of how plants adjust and balance the influences of different phytohormones is limited. Thus, in summary, the roles of phytohormones in AR formation are complex, and knowledge regarding the regulatory network is still scarce.

Among the endogenous factors that affect AR formation, reactive oxygen species (ROS) also play important roles in regulating AR formation. Generally, ROS are considered signaling molecules and have been reported to regulate stomatal movements and biotic and abiotic stress responses as well as many aspects of plant development, including lateral root formation [16–20]. Recently, some studies have focused on the roles of ROS in AR formation. In mung bean, hydrogen peroxide (H_2_O_2_), one of the main types of ROS, has been predicted by RNA-Seq analysis to positively regulate AR formation by modulating a number of genes [21]. On olive adventitious rooting, an AOX protein (key enzyme on the alternative respiratory pathway) was found to putatively regulate the ROS homeostasis during the first stages of the adventitious rooting process [22]. Exogenous application of H_2_O_2_ has also been shown to increase the ability of marigold to form adventitious roots [23, 24]. Compared with that of other endogenous factors, the understanding of the role of ROS in AR formation is quite limited.

Poplar is a model tree species due to the small size of its genome, the early release of a high-quality draft genome and the availability of a highly efficient transgenic platform. Usually, poplar is considered as easy-rooting plant, however, different types of species still varied for this trait [25]. Recently, several studies have been performed on poplar AR formation. For example, transcripts involved in root and AR development were uncovered by expressed sequence tags sequencing [26]. The cytokinin type-B response regulator *PtRR13* was also found to act as a negative regulator of adventitious root development in *Populus* [27]. Subsequently, a transcription factor in the AP2 family was also found to play a role during AR formation [28]. Several WUSCHEL-related HOMEOBOX (WOX) transcription factors have also been reported to regulate key developmental processes in AR formation in poplar [29, 30]. However, there is still insufficient understanding of poplar AR formation compared with that of AR formation in herbaceous model plants. Here, in order to increase our understanding to the regulation of poplar ARs formations and screen of genes or endogenous factors that are responsible for this biological processes, we investigated the kinetics of poplar AR development and the dynamic gene expression during AR formation by RNA-Seq based transcriptomic analysis in poplar. Based on these data, the expression patterns of phytohormone-related genes were investigated, and their influences on AR formation were considered. Subsequent gene expression cluster analysis showed that genes involved in H_2_O_2_ pathways may contribute to poplar AR formation. Finally, pharmacological assay confirmed the influence of H_2_O_2_ on AR formation in poplar. Thus, in this study, we provide novel information on the regulation of AR formation in tree species.

## Results

### Poplar AR formation in WPM

**Fig. 1:**
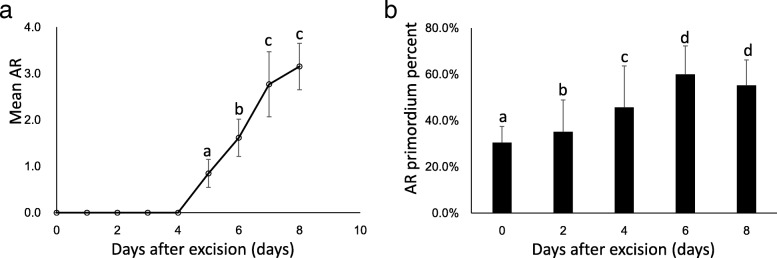
The kinetics of poplar AR development from DAE0 to DAE8 (**a**), mean AR from DAE0 to DAE8; (**b**), mean AR primordium percent investigated by anatomy. At least the basal region of 15 plantlets was collected for each time point and at least 10 cross sections (> 50 cross sections were prepared and 10 were randomly selected) were used per cutting. The AR percentage was calculated considering the number of sections with AR primordia in each cutting in comparison with the total number of sections analyzed per cutting. Significant difference among multiple comparisons were labeled as different letters above the dot in **a** and bar in **b** (*P* = 0.05)

**Fig. 2:**
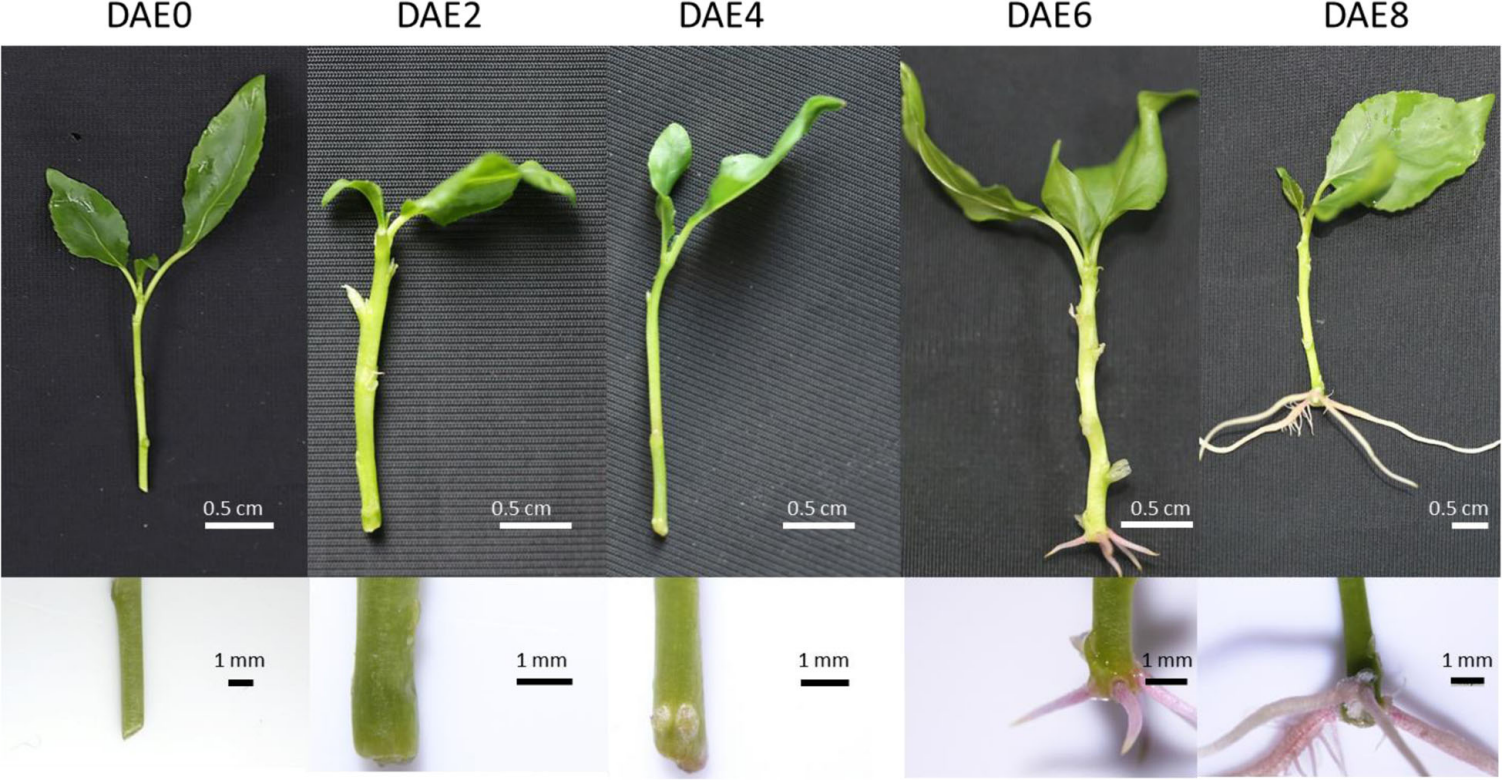
AR development from DAE0 to DAE8 in WPM

To investigate the kinetics of poplar AR formation, 30 microcuttings with 2–3 upper leaves and an apical bud (ca. 2–3 cm) of “NL895” were cultured in WPM in the absence of plant hormones and other growth regulators. The first visible AR was observed at 5 days after excision (DAE5), and > 80% of the microcuttings showed roots at DAE8. The number of ARs from DAE0 to DAE8 is shown in Fig. 1a. There were no visible ARs from DAE1 to DAE4, while 0.8 ± 0.3, 1.6 ± 0.4, 2.8 ± 0.7 and 3.2 ± 0.5 ARs could be observed at DAE5, DAE6, DAE7, and DAE8, respectively. After DAE8, the total number of ARs increased slightly, and the length of the ARs increased sharply (data not shown). The typical AR formation processes from DAE0 to DAE8 are illustrated in Fig. 2. According to the data showed in Figs. 1a and 2, it is easy to conclude that AR development mainly occurs inside stem cuttings at the first 4 days; while visible AR elongation mainly occurs from DAE4 to DAE8 for poplar cuttings growing in WPM.

**Fig. 3:**
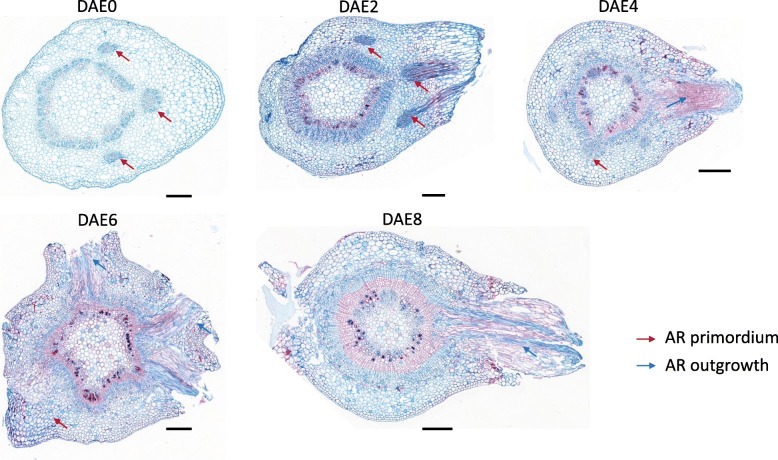
Anatomy of AR formation from in WPM from DAE0 to DAE8 Bar = 20 μm

When using anatomical method to investigate poplar AR formation in WPM, we found there were different AR primordia rate among the 5 tested stages. The percent are DAE0 (30.4% ± 7.0%), DAE2 (35.2% ± 13.7%), DAE4 (45.7% ± 17.9%), DAE6 (60.0% ± 12.3%) and DAE8 (55.3% ± 11.0%) (Fig. 1b). With multiple comparison (*P* = 0.05), significant difference among DAE0, DAE2 and DAE4; while difference between DAE6 and DAE8 didn’t reach significant level. The typical sections from DAE0 to DAE8 are illustrated in Fig. 3. Clearly, a relative high rate of section could be observed AR primordia in stem cortex at DAE0 (30.4 ± 7.0%), it suggested that there were AR primordia formed before the preparation of cuttings. Additionally, AR primordia rate also increased after DAE0 (except DAE8, the slightly decreased percent might be attributed to difficulty in counting primordia due to large number of mature AR), it suggested that poplar cuttings also could form new AR primordia during its development. Therefore, both pre- and newly- formed AR primordia exist in poplar cuttings.

### Time course of gene expression during AR formation

To determine the gene expression profiles during AR formation, the 0.5-cm cutting bases inserted in the WPM (DAE0, DAE2, DAE4, DAE6 and DAE8) were collected for each time point. In total, 15 RNA-Seq libraries were created (three biological samples for each time point) using the collected samples. A total of approximately 284 million 150-bp paired-end (PE) reads were obtained (Table 1). After quality control and filtering, 84.0 G of clean data remained, and the average amount of data in the samples was 5.60 ± 0.61 G (Table 1). The smallest sample was DAE4–1 with 4.83 G, while the largest sample was DAE2–2 with 6.29 G of clean data (Table 1). The clean reads constituted 98.5% of all raw reads. The high quality and sufficient quantity of the RNA-Seq data supported further gene expression analysis.

**Table 1:** Statistical summary of RNA-Seq libraries

Libraries	Raw Reads	Clean Reads	Mapped	%Mapped
DAE0–1	20,369,527	20,063,000	14,284,856	71.2%
DAE0–2	20,367,950	20,063,416	14,545,977	72.5%
DAE0–3	20,369,457	20,063,916	14,024,677	69.9%
DAE2–1	21,298,025	20,975,600	13,697,067	65.3%
DAE2–2	21,297,125	20,975,699	13,718,107	65.4%
DAE2–3	21,297,237	20,975,809	13,319,639	63.5%
DAE4–1	16,365,874	16,118,416	10,847,694	67.3%
DAE4–2	16,368,443	16,117,992	10,686,229	66.3%
DAE4–3	16,357,254	16,110,911	10,149,874	63.0%
DAE6–1	20,085,139	19,783,862	13,591,513	68.7%
DAE6–2	20,084,935	19,783,661	13,571,591	68.6%
DAE6–3	20,084,110	19,781,864	13,194,503	66.7%
DAE8–1	16,595,208	16,346,280	11,393,357	69.7%
DAE8–2	16,595,386	16,345,471	11,229,339	68.7%
DAE8–3	16,594,200	16,345,287	10,706,163	65.5%

All the clean reads were mapped onto the reference genome, and the gene expression numbers were calculated by TopHat and Cufflinks [31, 32]. To identify differentially expressed genes, gene expression at the four later time points, DAE2, DAE4, DAE6 and DAE8, was compared with that at DAE0. Moreover, consecutive time points were also compared, including DAE2 and DAE4, DAE4 and DAE6, and DAE6 and DAE8. Therefore, a total of 7 paired comparisons were conducted. According to the criteria for differentially expressed genes mentioned in the Materials and Methods section, there were 7483 nonredundant differentially expressed genes (Additional file 1: Table S2). When these differentially expressed genes were subjected to cluster analysis, samples from the same time point clearly tended to cluster together (Additional file 2: Figure S1). These results suggested that the samples collected were of high quality. Moreover, DAE8 and DAE0 showed less distance than the other time points and could be grouped in one subclade, while DAE2, DAE4, and DAE6 were in another subclade. The existence of 2 expression subclades is consistent with the above result regarding the kinetics of poplar AR formation. AR formation mainly occurred from DAE2 to DAE6, while the stem base returned to normal growth by DAE8.

When the differentially expressed genes for all the selected comparisons were examined, there were 4882, 4774, 4354, 3129, 940, 1173 and 2155 genes showing differential expression between DAE0 and DAE2, DAE0 and DAE4, DAE0 and DAE6, DAE0 and DAE8, DAE2 and DAE4, DAE4 and DAE6, and DAE6 and DAE8, respectively (Additional file 3: Figure S2). The highest number of differentially expressed genes was found between DAE0 and DAE2, and the lowest number of differentially expressed genes was found between DAE2 and DAE4. Of the 4 consecutive comparisons (DAE0 and DAE2, DAE2 and DAE4, DAE4 and DAE6, and DAE0 and DAE8), DAE0 and DAE2 had the highest number of differentially expressed genes, while DAE2 and DAE4 had the lowest number. Since consecutive comparisons are likely reliable reflections of the dynamic changes in gene expression during AR development, this result might suggest that the period of time from 0 to 2 DAE is the most important stage for AR formation.

**Fig. 4:**
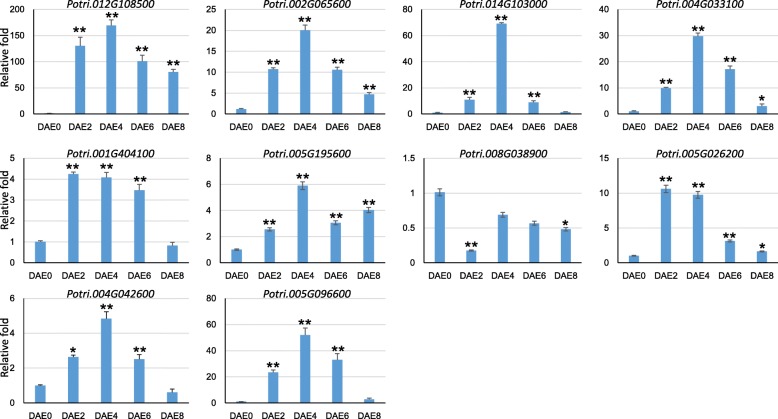
Gene expression analysis of the 10 selected genes by RT-qPCR assay. The FPKM values of 9 genes, excluding *Potri.001G404100*, can be found in Additional file 1: Table S2. The FPKM values of *Potri.001G404100* for DAE0, DAE2, DAE4, DAE6, and DAE8 are 23.1, 27.9, 23.6, 23.2 and 13.2, respectively. Because these genes were not classified as differentially expressed genes, their FPKM values are not in Additional file 1: Table S2. Two stars represent the relative expression fold is different between the tested time point and DAE0 under the significant level of *P* value equal to 0.01; while one star represents the relative expression fold is different between the tested time point and DAE0 under the significant level of *P* value equal to 0.05

To validate the gene expression data from RNA-Seq analysis, 10 genes showing patterns of differential expression among the 5 time points were randomly selected for RT-qPCR assay. The relative fold changes between each time point and the control samples (DAE0) were calculated by setting expression at DAE0 equal to 1 (Fig. 4). To compare the gene expression profiles of the RNA-Seq and RT-qPCR data, a correlation analysis was conducted. The two types of expression data were highly consistent, with an R^2^ value equal to 0.86 and a *p*-value less than 0.0001. This high R^2^ value clearly suggested that the expression results were very reliable for further analyses.

### Investigation of the expression patterns of phytohormone-related genes

Phytohormones are regarded as key regulators of AR formation. During AR formation, different hormones interact with each other and form a large and complex network that regulates this dynamic biological process. To gain insight into the roles of phytohormones in AR formation, we investigated phytohormone-related genes in poplar with homology searching (see Materials and Methods). In total, 807 genes were predicted to be involved in biosynthesis, transport, metabolism and reception of the 8 phytohormones (Additional file 4: Table S3). These 8 phytohormones included ABA, auxin, BRs, cytokinins, ET, GAs, JA and SA, and the corresponding numbers of related genes were 75, 201, 291, 29, 42, 58, 82, and 29, respectively (Table 2). No genes were predicted to be involved in BR transport, ET transport and metabolism, GA transport and metabolism, JA transport and SA reception (Table 2). When the expression patterns for these phytohormone-related genes were examined, 180 genes showed differential expression during AR formation; their normalized expression values are shown in Additional file 5: Table S4.

**Table 2:** The number of phytohormone-related genes in poplar

Phytohormone	Biosynthesis	Transport	Metabolism	Receptor
ABA	6/40^a^	3/10	3/14	2/11
Auxin	0/14	50/174	4/7	1/6
BR	14/53	NA	10/54	30/184
Cytokinin	2/5	1/8	1/9	4/7
ET	6/35	NA	NA	2/7
GA	8/53	NA	NA	3/5
JA	23/78	0/2	NA	0/2
SA	0/1	0/2	7/26	NA

^a^The number in the denominator indicates the total number of genes predicted for the corresponding function, while the number in the numerator indicates the number of genes differentially expressed during AR formation. NA indicates that there are no genes in the category

**Fig. 5:**
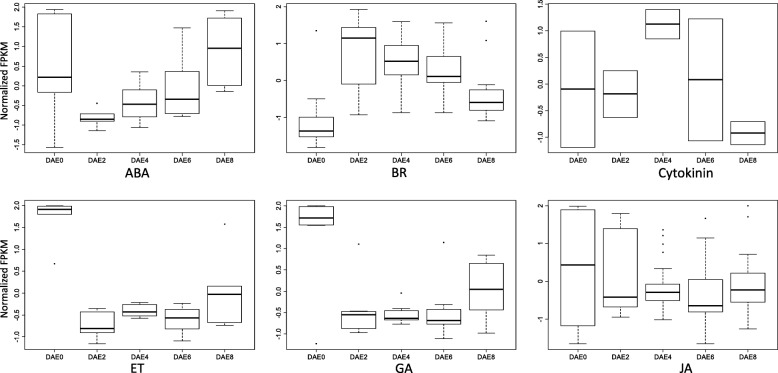
Boxplot of FPKM values for differentially expressed genes related to phytohormone biosynthesis. FPKM represents fragments per kilobase of transcript per million fragments mapped. Each boxplot consists of the Q1 (1/4 quartiles, the bottom of the box), Q3 (3/4 quartiles, the top bottom of the box), the median Q2 (the line inside into the box), Q3 + 1.5 IQR (interquartile range, Q3-Q1, the highest line outside the box) and Q1–1.5 IQR (the lowest line outside the box). Values larger than Q3 + 1.5 IQR or less than Q1–1.5 IQR were considered as outliers and they were plotted as dots outside of the Q3 + 1.5 IQR or Q1–1.5 IQR. If Q3 + 1.5 IQR is less than Q3 or Q1–1.5 IQR is less than Q1, their corresponding lines will not be shown. ABA represents abscisic acid, BR represents brassinosteroids, ET represents ethylene, GA represents gibberellins and JA represents jasmonic acid

We therefore summarized the dynamic gene expression that occurred over the 5 AR time points. Among the phytohormone biosynthesis genes, clear patterns could be observed for 6 hormones (Fig. 5). No auxin and SA biosynthesis genes showed differential expression during AR formation. The expression of ABA biosynthesis genes was reduced at DAE2, DAE4 and DAE6 and was then slightly elevated to normal levels by DAE8. The expression of BR biosynthesis genes increased sharply to a very high level by DAE2 and then slightly decreased at subsequent time points; however, the expression of these genes from DAE2 to DAE8 remained higher than at DAE0. In contrast, the expression of ET and GA biosynthesis genes showed an opposite trend. Gene expression in this category decreased sharply to a very low level by DAE2 and then slightly increased at subsequent time points. Cytokinin biosynthesis genes showed the highest and lowest expression at DAE4 and DAE8, respectively, while their expression remained at normal levels at the other 3 time points. The expression of JA biosynthesis genes was reduced at all stages compared to that at DAE0.

Among the phytohormone transport genes, only 3 hormones, ABA, auxin and cytokinins, had data that enabled an investigation of their expression patterns (Additional file 6: Figure S3). The average expression of all 3 phytohormone transport genes was higher at DAE0 than other stages. The plant material used for the stage of DAE0 also represents a tissue of a plant at normal growth status. Thus, the highest average expression for those phytohormone transport genes at DAE0 suggested that transport of ABA, auxin and cytokinins is required for poplar normal growth. The transport of the 3 phytohormones might also support the requirement of the initiation of AR development in poplar. At the stage of DAE2, the average expression of these phytohormone transport genes decreased sharply might be attributed to the excision of shootings from mother plants. The steadily increase of the average expression of these phytohormone transport genes from DAE2 to DAE8 suggested that ABA, auxin and cytokinins were involved more and more for poplar AR development. Since the processes of biosynthesis and transport are the source of endogenous phytohormones in plants, the dynamic expression patterns of these 7 phytohormone biosynthesis and transport genes might reflect their roles during AR formation.

Among the phytohormone metabolism genes, those related to BRs, cytokinins and SA had elevated expression from DAE2 to DAE8 compared to their expression at DAE0 (Additional file 7: Figure S4). Among the phytohormone receptor genes, those related to 6 hormones had data that enabled an investigation of their expression patterns (Additional file 8: Figure S5). BR and cytokinin receptor genes showed similar expression patterns, decreasing sharply by DAE2 and then increasing at subsequent stages; however, their expression at DAE8 remained lower than at DAE0. ABA and GA receptor genes also shared similar expression patterns, which demonstrated trends opposite those of the BR and cytokinin receptor genes. Auxin and ET receptor genes also showed opposite patterns to those of the BR and cytokinin receptor genes; the expression of auxin receptor genes first decreased at DAE2 and DAE4 and then increased by DAE6 and DAE8 to a higher level than at DAE0. Usually, both negative and positive regulation exists for metabolism and receptor genes when they respond to the corresponding phytohormones, therefore, we can’t conclude roles for these genes during poplar AR formation.

### Functional annotation of genes differentially expressed during AR formation by gene clustering

**Fig. 6:**
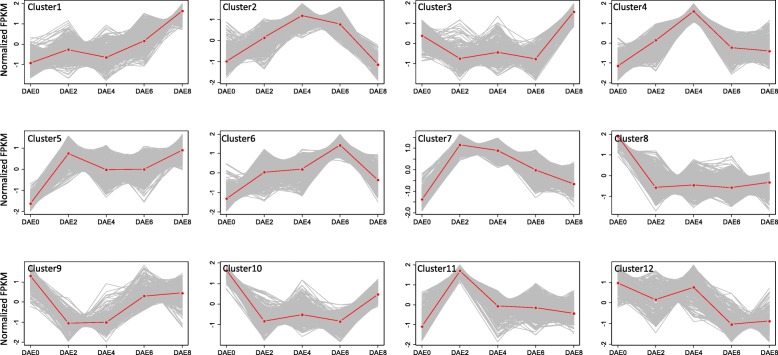
Clustering of genes differentially expressed during AR formation. FPKM represents fragments per kilobase of transcript per million fragments mapped

To comprehensively investigate other factors affecting AR formation in poplar, we classified the differentially expressed genes by clustering them based on their dynamic expression patterns. With the K-means algorithm, 12 clusters were formed for these 7483 differentially expressed genes. There were 551, 743, 304, 479, 457, 454, 854, 1567, 305, 896, 638 and 235 genes in clusters 1, 2, 3, 4, 5, 6, 7, 8, 9, 10, 11 and 12, respectively (Additional file 1: Table S2). The expression profiles for each cluster are shown in Fig. 6. The genes in clusters 1, 4, 5, 6 and 7 showed upregulation from DAE2 to DAE8 compared to their expression at DAE0, while the genes in clusters 8, 9, 10 and 12 showed downregulation at all time points compared to their expression at DAE0 (Fig. 6). However, all 12 clusters had unique expression patterns, with the highest and lowest mean expression values at different AR formation stages. The large number of clusters and the diverse expression patterns suggest that AR formation in poplar is a complex biological process.

The grouping of genes in the same cluster usually indicates that those genes are involved in similar biological processes. Therefore, functional enrichment analyses were performed for each gene cluster. Ten of the 12 clusters showed enrichment of at least one biological function (Additional file 9: Table S5); only clusters 3 and 9 failed to show any enrichment. The most enriched biological process for cluster 1 was “photosynthesis”, and the most enriched process for cluster 5 was “photosynthesis, light harvesting”. The gene expression patterns in these 2 clusters showed steady upregulation (Fig. 6). These results might suggest that photosynthesis took place during AR formation and that photosynthesis would be beneficial for poplar AR formation. Additionally, “chitin catabolic process”, “response to biotic stimulus”, and “defense response” were enriched in clusters 7 and 11. Previously, genes with these functions have been reported to be involved in plant responses to disease [33, 34]. Both clusters 7 and 11 showed peak expression at DAE2 before the expression gradually decreased to normal levels; this result might suggest that some disease response genes also play roles during AR formation. Since KEGG analyses more directly indicate gene function and their involved pathways, we carefully examined results from KEGG analysis for each cluster. In total, five KEGG pathways were enriched in 3 gene clusters (Table 3). “Tubulin beta” was enriched in cluster 2; this result is similar to the result described above for cluster 2 [35, 36]. Three KEGG pathways, “peroxidase”, “aquaporin PIP” and “aconitate hydratase 1/homoaconitase”, were enriched in cluster 6. “Large subunit ribosomal protein L39e” was enriched in cluster 12. Among these 4 KEGG pathways, “peroxidase” is usually responsible for ROS homeostasis, and ROS usually act as signaling molecules during plant development. Therefore, genes encoding peroxidase could possibly be involved in AR formation in poplar.

**Table 3:** Functional enrichment for gene clusters by KEGG analysis

Pathway	Cluster	Gene ID	DAE0	DAE2	DAE4	DAE6	DAE8
Tubulin beta	2	Potri.003G125400	−0.55 ^a^	− 0.55	1.38	0.96	− 1.25
		Potri.001G106100	−0.51	−0.33	1.35	0.91	−1.41
		Potri.001G246900	−0.90	−0.25	1.58	0.67	−1.09
		Potri.009G067100	−0.53	−0.45	0.99	1.32	−1.34
		Potri.009G040200	−1.10	0.26	1.13	0.95	−1.24
		Potri.002G021800	−0.55	−0.61	0.80	1.52	−1.17
Peroxidase	6	Potri.005G195600	−1.91	0.65	0.19	0.94	0.14
		Potri.003G214900	−1.71	−0.52	0.44	0.92	0.87
		Potri.007G122100	−0.49	−0.66	0.57	1.68	−1.09
		Potri.002G065300	−1.71	0.09	0.37	1.38	−0.13
		Potri.001G013000	−1.57	0.76	0.36	1.15	−0.70
		Potri.007G019300	−1.26	0.08	−0.10	1.77	−0.49
		Potri.006G129900	−0.99	−0.56	− 0.62	1.78	0.38
Aquaporin PIP		Potri.006G128200	−0.93	−0.31	− 0.30	1.95	− 0.40
		Potri.009G013900	−1.32	−0.34	0.25	1.73	−0.31
		Potri.006G128000	−0.97	−0.39	− 0.20	1.93	− 0.37
		Potri.016G113300	−0.79	−0.88	0.43	1.77	−0.52
Aconitate hydratase 1/homoaconitase		Potri.014G153400	−1.47	−0.17	0.85	1.34	−0.54
		Potri.005G108100	−1.62	0.67	0.24	1.25	−0.54
		Potri.002G229200	−1.75	0.24	0.46	1.27	−0.23
Large subunit ribosomal protein L39e	12	Potri.018G022100	0.20	0.71	1.12	−0.25	−1.77
		Potri.006G260500	0.63	0.44	1.27	−0.99	−1.35
		Potri.018G112300	0.47	0.17	1.10	0.14	−1.88

^a^All the numbers are the normalized FPKM values

### Investigation of the roles of H_2_O_2_ in poplar AR formation

**Fig. 7:**
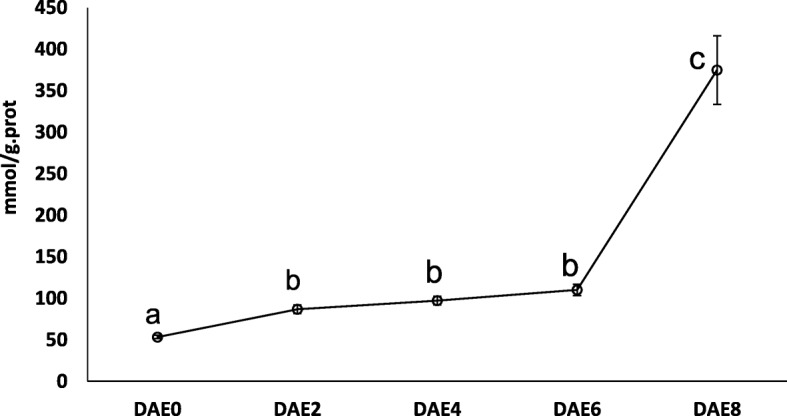
H_2_O_2_ accumulation during AR formation. Five to 15 stem bases were harvested to form one biological samples (at least 0.1 g) and 6 biological samples were prepared for one treatment. The multiple comparisons was performed under the significant level of P value equal to 0.05. The error bar represents standard deviation for each treatment

To test the above hypothesis, we mainly focused on genes in the peroxidase pathway. A total of 7 differentially expressed genes encoding peroxidase were grouped in cluster 6, and most of these genes showed increased expression after DAE0 and peak expression at DAE6 (Table 3). The gene *Potri.005G195600* was selected for RT-qPCR assay. The results were very consistent with the RNA-Seq results (Fig. 4). After RT-qPCR analysis, the H_2_O_2_ content in the basal stems of tissue culture plantlets was tested. H_2_O_2_ accumulation clearly increased during the entire process of AR formation. The H_2_O_2_ content was 53.5 ± 2.0, 86.8 ± 5.2, 97.3 ± 5.3, 110.2 ± 6.7 and 374.8 ± 41.3 mmol/g.prot for DAE0, DAE2, DAE4, DAE6, and DAE8, respectively (Fig. 7). Through variance analysis and multiple comparisons, the content of H_2_O_2_ at DAE2, DAE4 and DAE6 was revealed to be significantly higher than at DAE0 (*P* < 0.05), while the H_2_O_2_ content at these 3 time points was significantly lower than at DAE8 (*P* < 0.05). Therefore, we concluded that the H_2_O_2_ content showed three levels during AR formation: the H_2_O_2_ content was at a baseline level at the beginning time point; it increased and remained at a relatively high level from DAE2 to DAE6, and it then increased sharply at DAE8.

**Fig. 8:**
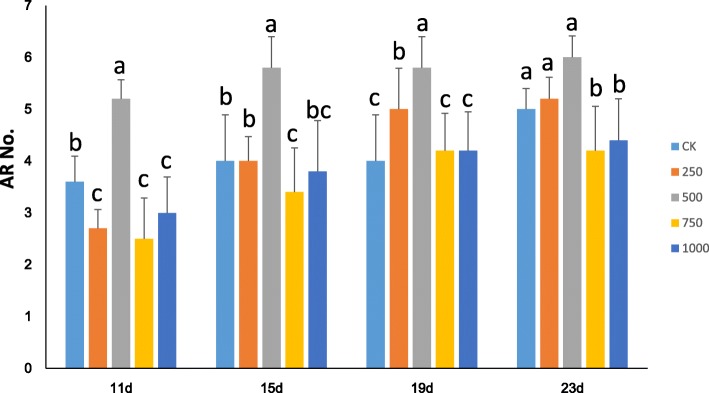
AR numbers during hydroponic culture of poplar cuttings with different concentrations of supplemented H_2_O_2_. There were 15 plants for 1000 μM H_2_O_2_ treatment; while 30 plants for all other treatments. AR number was presented by mean value of each treatment. The multiple comparisons was performed under the significant level of *P* value equal to 0.05. The error bar represents standard deviation for each treatment

**Fig. 9:**
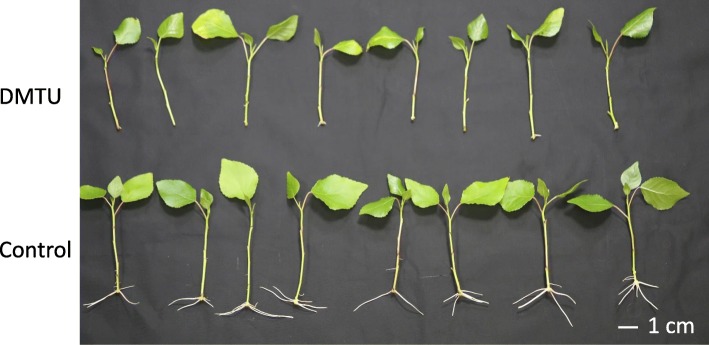
Aspect of poplar cuttings 12 days after establishment in hydroponic culture with or without DMTU

**Fig. 10:**
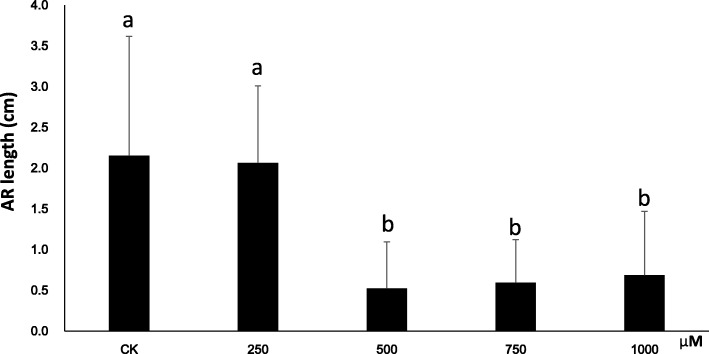
AR length of poplar cuttings 12 days after establishment under hydroponic culture with different concentrations of H_2_O_2.._ The plant number for each treatment were the same as Fig. 8. AR length was presented by mean value of each treatment. The multiple comparisons was performed under the significant level of *P* value equal to 0.05. The error bar represents standard deviation for each treatment

To further understand the roles played by H_2_O_2_ during poplar AR formation, we performed hydroponic culture by using semi-hardwood cuttings of poplar “NL895” as plant material. In the preliminary experiment, five cuttings with uniform growth status were grown in hydroponic solution supplemented with 0, 500 or 1000 μM H_2_O_2_. Cuttings grown in 500 μM H_2_O_2_ produced markedly more ARs at DAE11 than cuttings grown in the other H_2_O_2_ concentrations (Additional file 10: Figure S6). Then, an average of 30 cuttings with uniform growth status were grown in each of 5 hydroponic solutions (Note, only 15 data were finally obtained for 1000 μM H_2_O_2_ treatment), and the number of ARs for each cutting was recorded every day. The number of ARs was recorded after 11, 15, 19 and 23 days of growth in hydroponic solution supplemented with 0, 250, 500, 750 or 1000 μM H_2_O_2_. The results showed that the average number of ARs was significantly affected by the concentration of H_2_O_2_ (Fig. 8). From days 11 to 19, the average AR number for the cuttings grown in the solution with 500 μM H_2_O_2_ was higher than that for the cuttings grown in the other solutions (*P* < 0.05), while the differences between the average AR numbers seemed to decline after day 19. Additionally, the average AR numbers of the cuttings grown in the solutions with 750 and 1000 μM H_2_O_2_ were lower than those of the cuttings grown in the untreated control solution (0 μM). Therefore, three findings were evident from these data. First, treatment with exogenous H_2_O_2_ could affect AR formation; second, AR developed faster in the 500 μM solution than in the other solutions; and third, higher concentrations of exogenous H_2_O_2_ slowed AR formation in poplar. All these results also suggest that appropriate levels of H_2_O_2_ production can accelerate AR formation in poplar. To validate the above results, poplar “NL895” cuttings were also grown in hydroponic solution with 0.5 mM DMTU, which is a chemical H_2_O_2_ scavenger. By day 8, the H_2_O_2_ content in the stem base of cuttings that were grown in DMTU solution was 142.7 ± 7.3 (*n* = 30), while that in the stem base of untreated controls was 367.7 ± 3.2 (*n* = 30). This revealed that DMTU reduced the accumulation of H_2_O_2_ (T test, *P* < 0.05). The average number of ARs in the cuttings grown in hydroponic solution with 0.5 mM DMTU was significantly lower than that in the cuttings grown in control solution at DAE12 (Fig. 9). The average number of ARs in DMTU was 0.8 ± 1.1, while that in the untreated controls was 6.6 ± 1.3. This result supports the finding that appropriate levels of H_2_O_2_ production can accelerate AR formation in poplar. Though AR developed faster in solutions with appropriate concentrations of H_2_O_2_, the influence of H_2_O_2_ on AR length seemed to follow a different trend. At day 23, the average AR lengths for the cuttings grown in the solutions supplemented with 0, 250, 500, 750 and 1000 μM H_2_O_2_ were 2.2 ± 1.5, 2.1 ± 0.9, 0.5 ± 0.6, 0.6 ± 0.5 and 0.7 ± 0.8 cm, respectively (Fig. 10). The solutions supplemented with 500, 750 and 1000 μM H_2_O_2_ had cuttings with significantly lower average AR lengths than the 2 solutions supplemented lower concentrations (*P* < 0.05). Although the average AR length was not significantly different between cuttings grown in solutions supplemented with 0 and 250 μM H_2_O_2,_ the absolute number of ARs was lower in cuttings from the solution with 250 μM H_2_O_2_. This result might suggest that H_2_O_2_ negatively regulates AR elongation in poplar.

## Discussion

### Complex biological processes occur in poplar AR formation

Poplars is considered as easy-rooting species and there is still complex biological processes during AR formation [1]. In previous studies, the development of poplar AR was classified into 3 stages: primordium organization, primordium differentiation and root elongation [4, 7]. As shown in Figs. 1b and 3, our results were partially consistent with this conclusion. Our data showed that primordium organization and differentiation also happened before the excision of cuttings. Both pre- and newly- formed primordia contribute to AR formation for poplar “NL895”. This result is consistent with previous reports [37–39]. Some cuttings produced ARs from DAE5 to DAE8, while others did not produce ARs over the same time points. Newly-formed AR primordium could also be observed at all 5 stages. This result suggested that there were no clear boundaries between stages and that the 3 processes (primordium organization, primordium differentiation and root elongation) might exist simultaneously at the same time point within one stem cutting after DAE4. However, the time points of the major developmental events were clear; primordium organization and differentiation mainly occurred from DAE0 to DAE4, and root elongation mainly occurred from DAE4 to DAE8. Therefore, we classified AR formation in poplar “NL895” into 2 stages, early and late. The early stage is from DAE0 to DAE4 and AR development occurs at the inside of stems; while the late stage is from DAE4 to DAE8 and AR development occurs at the outside of stems. According to the above analysis, we can conclude that 1) the key stage for AR formation in WPM is in the first 8 days after excision; 2) both pre- and newly- formed AR primordia contribute to AR formation for poplar; 3) the production of newly formed AR primordia occurs at most of the whole stage from DAE0 to DAE8. Therefore, our data demonstrated that the development of poplar ARs is a dynamic and complex biological process.

### Multiple factors show influence on poplar AR formation

Phytohormones are the most important factors affecting AR formation. Among diverse phytohormones, auxin has been confirmed to play a central role in AR formation in many studies [8–10]. Since transcriptomic analysis has been proved to be a powerful strategy for uncover underlying mechanisms for a given biological process, many studies have been focused on root or AR formation by using this method [22, 25, 26]. In this study, we also uncovered some underlying influencing factors for poplar AR regulation. According to data illustrated in Fig. 5 and Additional file 6: Figure S3, Additional file 7: Figure S4 and Additional file 8: Figure S5, there were no genes related to auxin biosynthesis showing differential expression during AR formation in this study; while 50 genes related to auxin transport showing differential expression. Therefore, this data suggested that there was no local biosynthesis occurring during poplar AR formation and the accumulation of auxin was mainly attributed to transport. This result is similar to previous reports [10]. According to the expression profiles of the phytohormone biosynthesis- and transport-related genes and the accumulation characteristics (local biosynthesis or transport from other sites), we were able to deduce the roles of 7 investigated phytohormones, including ABA, auxin, BRs, cytokinins, ET, GAs and JA. These 7 phytohormones could clearly be classified into 5 groups. The first group included only one phytohormone, ABA. Local ABA biosynthesis decreased from DAE2 to DAE6 and then increased by DAE8. This pattern suggested that ABA negatively regulates primordium organization and primordium differentiation but positively regulates root elongation during AR formation. The second group included one phytohormones, BRs. This phytohormone positively regulated all 3 AR developmental stages, but their effects decreased late in AR development. The third group included one phytohormone, cytokinins. The roles of cytokinins in AR regulation are complex compared to those of other phytohormones. The expression of cytokinin biosynthesis genes had very large variation from DAE0 to DAE8; the expression levels were slightly decreased by DAE2, sharply elevated by DAE4, and then decreased by DAE6 and DAE8. This complex expression profile suggested a complex role in which cytokinins act as positive regulators at some stages and as negative regulators at the other stages. The fourth group included 3 phytohormones, ET, GAs and JA. All of these phytohormones exerted negative effects on AR formation at all 3 stages. The fifth included one phytohormones, auxin. Its accumulation mainly depended on transport and it positively regulates AR formation at both early and late stages.

Besides phytohormones, other factors could be deduced to show influence on poplar AR formation according to the RNA-Seq results. According to the data listed in Additional file 9: Table S5, some of the results agreed strongly with those of previous studies. For example, the “microtubule” function was enriched in cluster 2 [35, 36], and “response to hormone” and “response to wounding” were enriched in clusters 10 and 11 [33, 34], respectively. Moreover, some interesting and novel information on poplar AR formation was also revealed. The photosynthesis and carbon fixation pathways enriched in gene expression cluster 1 and the expression of this cluster was steadily increased from DAE0 to DAE8 (Fig. 6 and Additional file 9: Table S5). This result might suggest that sugar-signaling pathway is involved in poplar AR formation. Similarly, wounding responsive, biotic stimulus and defense regulation pathways showed enrichment in cluster 11 and the expression of this cluster was sharply increased at DAE0 (Fig. 6 and Additional file 9: Table S5). The related gene expression was abundant at early stage of poplar AR formation might suggest that the excision of poplar cuttings activated wounding signal and subsequently inducted AR initiation. This prediction is very similar to previous reports [26]. According to result illustrated in Fig. 6 and Additional file 9: Table S5, more other factors that show potential involvement in poplar AR formation also could be found, such as secondary metabolites, energy, nitric oxide and redox. All these results are similar to a number of previous studies [23, 24, 40]. Therefore, we could conclude that multiple factors show influence on poplar AR formation.

### H_2_O_2_ could affect poplar AR formation

In this study, we investigated the influence of H_2_O_2_ on poplar AR formation by using pharmacological tests. The accumulation of H_2_O_2_ was increased after DAE0 (Fig. 7). However, we also could observe that H_2_O_2_ content at DAE8 was also significantly higher than DAE2 to DAE6 (*P* < 0.05). It suggested that there were 3 levels of H_2_O_2_ content from DAE0 to DAE8, with the lowest, moderate and highest at DAE0, DAE2 to DAE6 and DAE8, respectively. Since there were visible ARs at DAE8 and we couldn’t completely exclude the influence of newly formed ARs on H_2_O_2_ content assay, thus, the much higher H_2_O_2_ accumulation at DAE8 might be partially attributed to the newly formed ARs (poplar roots generated higher H_2_O_2_ content than stems, data not shown). However, the accumulation of H_2_O_2_ was still higher at DAE2 to DAE6 than at DAE0 (from 53.5 ± 2.0 to 110.2 ± 6.7 mmol/g.prot), it revealed that accumulation of H_2_O_2_ was indeed increased during the early stage of poplar AR formation. In previous studies, H_2_O_2_ was also found to be involved in AR or lateral root formation, such as in Mung bean [21], Marigold [23, 24] and Alfalfa [17]. Comparing with these studies, our result is highly consistent with previous reports.

The data clearly revealed that an appropriate content of H_2_O_2_ could accelerate the formation of AR, while higher H_2_O_2_ content could slow AR formation and reduce root elongation. H_2_O_2_ is a type of reactive oxygen species that has various roles in many biological processes, including responses to biotic and abiotic stresses and plant growth [40]. However, the underlying mechanisms of its influence on poplar AR formation are unclear. Some previous studies have reported a relationship between H_2_O_2_ and a number of phytohormones [41]. Here, we also predicted the roles of 7 phytohormones and H_2_O_2_ during poplar AR formation. Since the accumulation of H_2_O_2_ increased steadily during the whole AR formation process and since two phytohormones, auxin and BRs, positively regulated AR formation, these results might suggest that there was direct or indirect regulation among H_2_O_2_, auxin and BRs. In *A. thaliana*, ROS act as downstream regulators of auxin in the promotion of root hair development [42]. Based on our data, we could also speculate that H_2_O_2_ was regulated by auxin and BRs during AR formation. An appropriate H_2_O_2_ content promoted AR formation. However, there are 3 stages for poplar AR formation, primordium organization, primordium differentiation and elongating roots. The H_2_O_2_ concentrations at these 3 stages were significantly different (*P* < 0.05). Thus, we cannot conclude whether H_2_O_2_ plays a role at all 3 stages or only at a special stage. Therefore, more research should be performed to comprehensively determine the influence of H_2_O_2_ on AR formation in poplar.

## Conclusions

Here, we were able to provide a global view of the roles of phytohormones and H_2_O_2_ during poplar AR formation by using RNA-Seq and pharmacological testing. The results showed a large and complex regulatory network among diverse phytohormones and H_2_O_2_. The knowledge gained from this study will help us to understand the underlying mechanisms of AR formation.

## Methods

### Plant materials, growth conditions and treatments

A hybrid variety of poplar “NL895” (*Populus. euramericana*), which forms ARs easily both in tissue culture and field cultivation, was used as plant material in this study. This variety was obtained from the tree germplasm repository of Huazhong Agricultural University (HZAU) under legal permission. For the investigation of the kinetics of poplar AR formation and for the preparation of RNA for high-throughput sequencing, tissue culture plantlets of “NL895” were used as plant material. Woody plants with different juvenile periods exhibit very different AR-forming abilities; therefore, two strategies were employed to avoid this influence. First, all microcuttings with undifferentiated ARs were obtained from apical buds that were generated from the first subculture of the differentiated poplar explants. Second, only microcuttings with 2–3 upper leaves and an apical bud (ca. 2–3 cm) were propagated in woody plant medium (WPM) for further analyses. The WPM was prepared according to previous report [43]. Additionally, all microcuttings that were inserted similar deep into the WPM (ca. 0.5 cm). All the tissue culture experiments were conducted at 25 °C with a 16/8 h photoperiod. To prepare samples for RNA extraction, the 0.5 cm cutting base was collected from each plantlet. Ten to 15 cutting bases were used for one biological sample. Three biological samples were prepared for each time point. Samples were collected at 5 time points: 0, 2, 4, 6 and 8 days after the cuttings were inserted into WPM. These time points were termed DAE0, DAE2, DAE4, DAE6 and DAE8, respectively. At DAE0, the cuttings had not experienced any treatment but were simply excised from the explants.

### Test the influence of H_2_O_2_ and N,N′-dimethylthiourea (DMTU) on AR formation

An hydroponic system was conducted to test the influence of H_2_O_2_ and DMTU on poplar AR formation. For the preparation of poplar semi-hardwood cuttings used in hydroponic propagation, young plantlets growing under in vitro conditions, with 6 to 10 cm in height, were acclimatized and then transplanted into 1-L soil pots. These plantlets were then grown for approximately 1 month until the plants had 10–15 leaves. Cuttings with 2–4 upper leaves and an apical bud were taken from the 1-month-old poplar plants. The hydroponic solution was prepared with double-sterilized water.

For the test of the influence of H_2_O_2_ on poplar AR formation, cuttings were inserted into the hydroponic solution with 0, 250, 500, 750 or 1000 μM H_2_O_2_. For the test of the influence of DMTU on poplar AR formation, cuttings were inserted into the hydroponic solution with 0.5 mM or without DMTU.

The number of visible ARs was recorded for one month for both experiments. For data collection and analysis, the AR length and number were recorded for each individual cutting and then average values were generated for each treatment. All hydroponic was conducted at 25 °C with a 16/8 h photoperiod. The hydroponic solution was changed every 2 days. An average of 30 cuttings were grown for each treatment during hydroponic propagation supplemented with H_2_O_2_ and DMTU, except the 1000 μM H_2_O_2_ treatment had 15 final collected data.

### Anatomical and microscopic analysis

For histological analysis, the same method and growth condition as RNA-Seq preparation were employed for collecting the 0.5 cm cutting base. At least 15 cutting bases were collected for each time points. The collected tissues were fixed in FAA (50% ethanol, 5% glacial acetic and 4% formaldehyde) and then treated with protocol described in a previous study [34]. Sections (15 μm) were made with rotary microtome (Leica RM2255) and stained with Safranin (1%) and fast-green (1%). At least 10 cross sections for one 0.5 cm cutting base were randomly selected for AR primordium counting. All the sections were observed and photographed with an Olympus BX51 microscope. The software FV10-ASW 3.1 Viewer (Olympus Support) was used to export photographs.

### H_2_O_2_ content assay

For H_2_O_2_ content assay, we followed protocol described in Bio-101 with slightly modification [44]. Briefly, the stem bases that were inserted into WPM or hydroponic solution were collected. Five to 15 stem bases were harvested to form one biological samples (at least 0.1 g) and 6 biological samples were prepared for one treatment. The sample (0.1 g) was ground to powder in liquid nitrogen and suspended with 4 °C phosphate buffer with thoroughly vortex. The mixture was centrifuged at 12000 rpm at 4 °C. The supernatant was used to test concentration of H_2_O_2_ with Amplex Red hydrogen peroxide/peroxidase assay kit (Molecular Probes, USA, CAS: A22188) according to its introduction. Six technique replications were set for each biological sample.

### Isolation of total RNA and whole transcriptome analysis

Total RNA was isolated using an RNeasy Plant Mini Kit according to the manufacturer’s instructions (DP432, TIANGEN Biotech (Beijing) Co., Ltd., Beijing, China). The quality of all RNA samples was examined by performing agarose gel electrophoresis and examination of the A260/280 wavelength ratio. The RNA integrity number (RIN) of all samples were also tested by an Agilent Bioanalyzer 2100. All samples had sufficient quality were used for RNA-Seq library construction according to a previously described protocol [45]. The constructed libraries were sent for sequencing using a HiSeq 2500 platform.

### Bioinformatic analysis

The raw RNA-Seq reads were quality trimmed and quality filtered using Trimmomatic software with the default parameter settings [46]. The *P. trichocarpa* genome version 3.0 was used as the reference genome in this study [47]. Clean reads from each sample were mapped onto the reference genome using TopHat software with the default parameters [31, 32]. Values of fragments per kilobase million (FPKM) were obtained by the software Cufflinks, and the differential expression was analyzed by Cuffdiff [31, 32]. A false discovery rate (FDR) less than 0.05 and a fold change (FC) greater than 2.0 were set as the thresholds for differentially expressed genes. Differentially expressed genes with FPKM values less than 10 at all 5 time points were excluded from further analysis.

For the annotation of phytohormone-related genes in poplar, a homology searching strategy was employed. First, phytohormone-related genes in *A. thaliana* were obtained from the Arabidopsis Hormone Database (http://ahd.cbi.pku.edu.cn/) [48], and genes with functions related to biosynthesis, transport, metabolism and receptors were kept. Second, a BLAST search of poplar genes that were homologous with the retained Arabidopsis hormone genes was conducted by using the command “blastp” in a local BLAST database (ftp://ftp.ncbi.nlm.nih.gov/blast/executables/blast+/LATEST/). The predicted proteins from the *P. trichocarpa* genome version 3.0 were used as queries. BLAST hits with scores greater than 250 (E-values of approximately e^− 60^) were kept. The biological processes of the retained poplar query proteins were annotated based on the functions of their top hit counterparts in the Arabidopsis Hormone Database.

To cluster the differentially expressed genes, the z-score (zero-mean normalization) of each FPKM value was determined with the “scale” command in R software (https://www.r-project.org/). The z-scores of all the differentially expressed genes were then used to form clusters with the “k-means” command in R software. The number of clusters was determined as previously described [49]. Functional annotation of the differentially expressed genes in each cluster was performed online with PlantGSEA (Plant GeneSet Enrichment Analysis Toolkit, http://structuralbiology.cau.edu.cn/PlantGSEA/) [50].

### Gene expression analysis

Ten differentially expressed genes were selected for RT-qPCR assay. The primers for these 10 randomly selected differential expressed genes are listed in Additional file 11: Table S1. RNA samples used for RNA-Seq analysis were used for gene expression studies. The concentration of each RNA sample was quantified with NanoDrop 8000 (Thermo Fisher Scientific CN, Shanghai, China) and equal amount of RNA was used for cDNA synthesis. A One-Step gDNA Removal and cDNA Synthesis SuperMix kit (AU311–02; Trans Biotech, Beijing, China) was used for cDNA synthesis. The PCR products of the 10 primer pairs were sent for performing Sanger sequencing to confirm amplification of the expected amplicons before the conduct of RT-qPCR assay. A LightCycler 96 (Roche) platform and FastStart Essential DNA Green Master mix (Roche Molecular Systems, Inc., China) were used for all RT-qPCR assays. Three technical replicates were performed for each sample. The standard curves for each primer pairs were examined to determine the amplification efficiency of a certain gene. Only primer pairs that showed the expected amplicons, PCR efficiency estimate value from 1.9 to 2.1 and unique melting curves (see below) were used for gene expression analysis. The cycling conditions were set as followed: 95 °C for 10 min and 40 cycles of 95 °C for 10 s and 65 °C for 60 s. Fluorescence data were collected during the 65 °C step and analyzed with the LightCycler 96 Manager. After the running of cycling, melting curves were also examined by an addtiontional program of 95 °C for 10 s, 65 °C for 60 s and 97 °C for 1 s. Sample quantification cycle (Cq) values were determined and were standardized relative to the expression of the two reference genes (ACTIN and UBQL, Additional file 11: Table S1). The 2^–ΔΔCq^ method was used to calculate the relative gene expression based on the RT-qPCR data [51]. The expression level at DAE0 for each tested gene was set as 1. All data analysis was performed by LightCycler® 96 Software (Version 1.1.0.1320, Roche, Shanghai, China).

### Statistical analysis

All the Statistical analysis was conducted by using the R software (https://www.r-project.org/). The function ‘aov’ was used for variance analysis and all multiple comparisons was performed under the significant level of *P* value equal to 0.05.

## Additional files

Additional file 1: **Table S2.** Genes differentially expressed during poplar AR formation. The numbers indicate FPKM values. (XLSX 507 kb)

Additional file 2: **Figure S1.** Dendrogram of RNA-Seq samples used for time course transcriptome analysis. (PPTX 125 kb)

Additional file 3: **Figure S2.** Number of genes that are differentially expressed during poplar AR formation. (PPTX 9022 kb)

Additional file 4: **Table S3.** Phytohormone-related genes in poplar (XLSX 30 kb)

Additional file 5: **Table S4.** Normalized expression values of differentially expressed phytohormone-related genes (XLSX 28 kb)

Additional file 6: **Figure S3.** Boxplot of FPKM values for differentially expressed genes related to phytohormone transport. FPKM represents fragments per kilobase of transcript per million fragments mapped. Each boxplot consists of the Q1 (1/4 quartiles, the bottom of the box), Q3 (3/4 quartiles, the top bottom of the box), the median Q2 (the line inside into the box), Q3 + 1.5 IQR (interquartile range, Q3-Q1, the highest line outside the box) and Q1–1.5 IQR (the lowest line outside the box). Values larger than Q3 + 1.5 IQR or less than Q1–1.5 IQR were considered as outliers and they were plotted as dots outside of the Q3 + 1.5 IQR or Q1–1.5 IQR. If Q3 + 1.5 IQR is less than Q3 or Q1–1.5 IQR is less than Q1, their corresponding lines will not be shown. (PPTX 38 kb)

Additional file 7: **Figure S4.** Boxplot of FPKM values for differentially expressed genes related to phytohormone metabolism. The figure legends are similar to Additional file 3: Figure S3. (PPTX 44 kb)

Additional file 8: **Figure S5.** Boxplot of FPKM values for differentially expressed genes related to phytohormone receptors. The figure legends are similar to Additional file 7: Figure S3. (PPTX 45 kb)

Additional file 9: **Table S5.** Functional enrichment of genes in the 12 clusters. (XLSX 15 kb)

Additional file 10: **Figure S6.** Hydroponic culture of poplar cuttings with different concentrations of supplemented H_2_O_2_ at DAE11. Panels a, b, and c represent poplar cuttings grown in 0, 500, and 1000 μM H_2_O_2_, respectively. Bar = 1 cm. (PPTX 579 kb)

Additional file 11: **Table S1.** Primers used for RT-qPCR assay. (XLSX 9 kb)

## Abbreviations

ABA: Abscisic acid
AR: Adventitious root
BR: Brassinosteroid
DAE: Days after excision
DMTU: Dimethylthiourea
ET: Ethylene
FPKM: Fragments per kilobase million
GA: Gibberellin
JA: Jasmonic acid
PAT: Polar auxin transport
ROS: Reactive oxygen species
SA: Salicylic acid
