# Supplementary figures and images for: Comprehensive analysis of dynamic gene expression and investigation of the roles of hydrogen peroxide during adventitious rooting in poplar
Source: BMC Plant Biol. 2019 Mar 12;19:99. doi: 10.1186/s12870-019-1700-7 (PMC6416884; doi:10.1186/s12870-019-1700-7)

## Slide 1
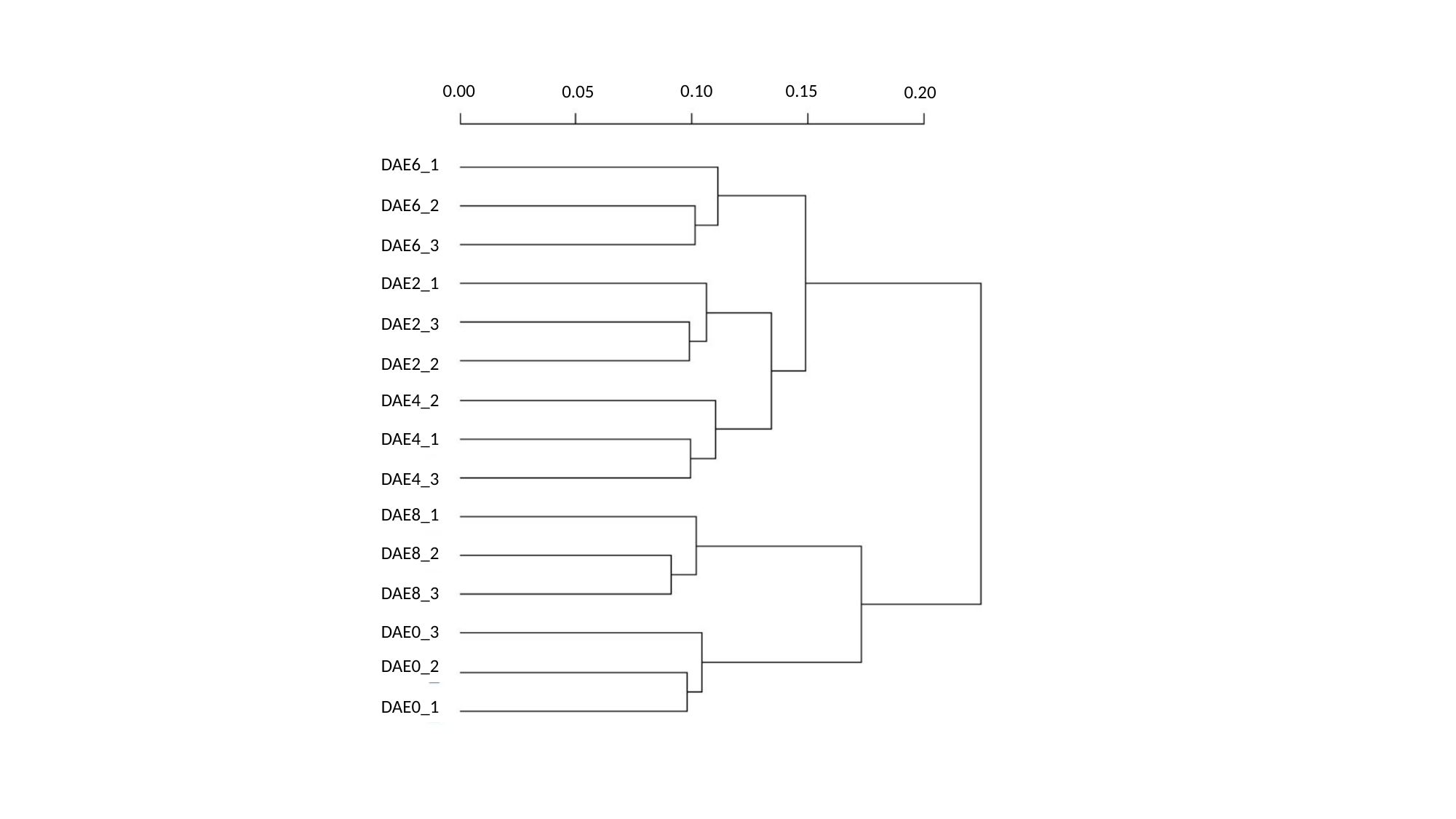

0.15
0.00
0.10
0.05
0.20
DAE6_1
DAE6_2
DAE6_3
DAE2_1
DAE2_3
DAE2_2
DAE4_2
DAE4_1
DAE4_3
DAE8_1
DAE8_2
DAE8_3
DAE0_3
DAE0_2
DAE0_1

Supplement: Supplementary file 2 — Figure S1. Dendrogram of RNA-Seq samples used for time course transcriptome analysis. (PPTX 125 kb) [file 12870_2019_1700_MOESM2_ESM.pptx]

## Slide 1
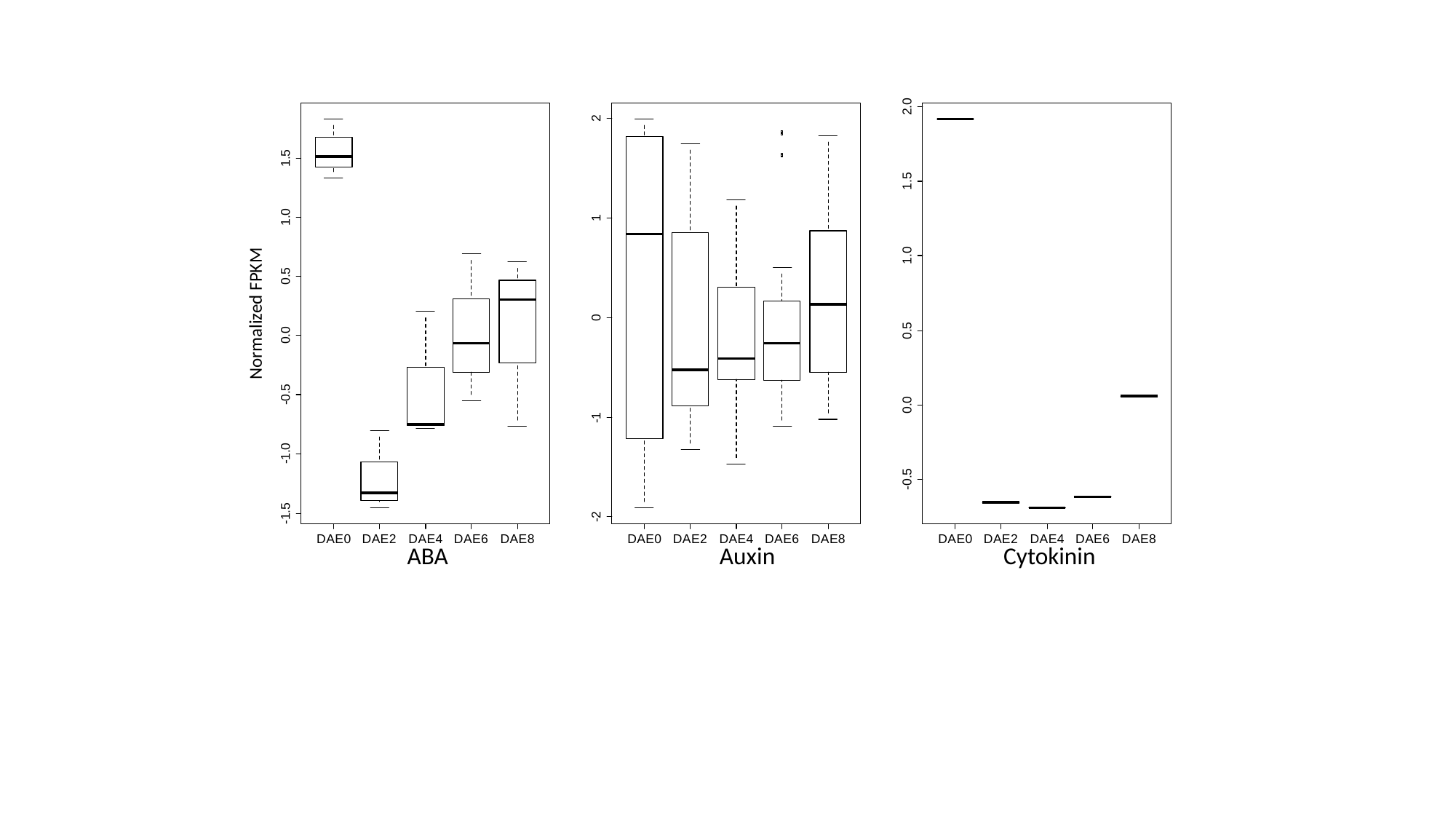

Normalized FPKM
ABA
Auxin
Cytokinin

Supplement: Supplementary file 6 — Figure S3. Boxplot of FPKM values for differentially expressed genes related to phytohormone transport. FPKM represents fragments per kilobase of transcript per million fragments mapped. Each boxplot consists of the Q1 (1/4 quartiles, the bottom of the box), Q3 (3/4 quartiles, the top bottom of the box), the median Q2 (the line inside into the box), Q3 + 1.5 IQR (interquartile range, Q3-Q1, the highest line outside the box) and Q1–1.5 IQR (the lowest line outside the box). Values larger than Q3 + 1.5 IQR or less than Q1–1.5 IQR were considered as outliers and they were plotted as dots outside of the Q3 + 1.5 IQR or Q1–1.5 IQR. If Q3 + 1.5 IQR is less than Q3 or Q1–1.5 IQR is less than Q1, their corresponding lines will not be shown. (PPTX 38 kb) [file 12870_2019_1700_MOESM6_ESM.pptx]

## Slide 1
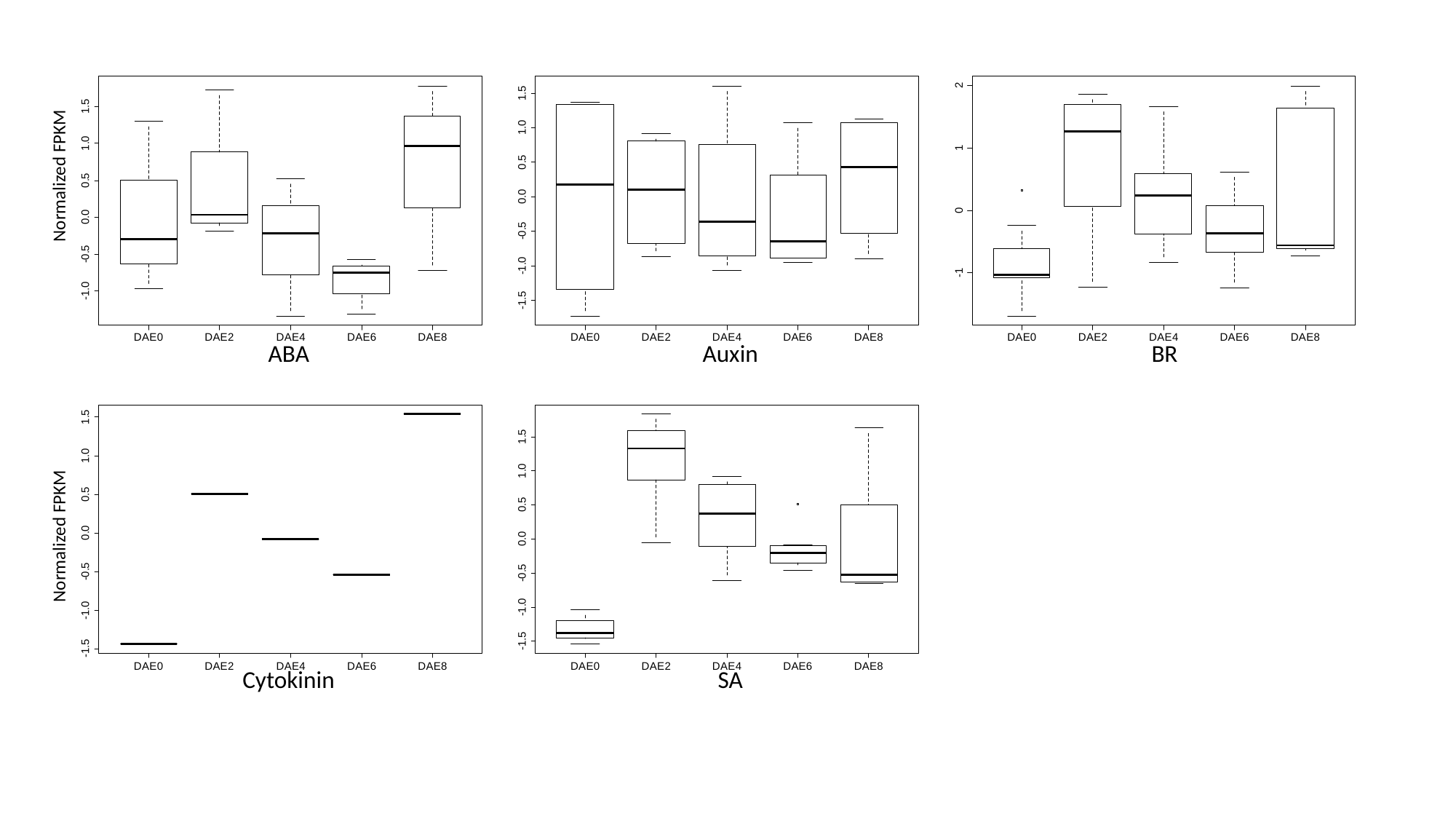

ABA
Auxin
BR
Cytokinin
SA
Normalized FPKM
Normalized FPKM

Supplement: Supplementary file 7 — Figure S4. Boxplot of FPKM values for differentially expressed genes related to phytohormone metabolism. The figure legends are similar to Additional file 3: Figure S3. (PPTX 44 kb) [file 12870_2019_1700_MOESM7_ESM.pptx]

## Slide 1
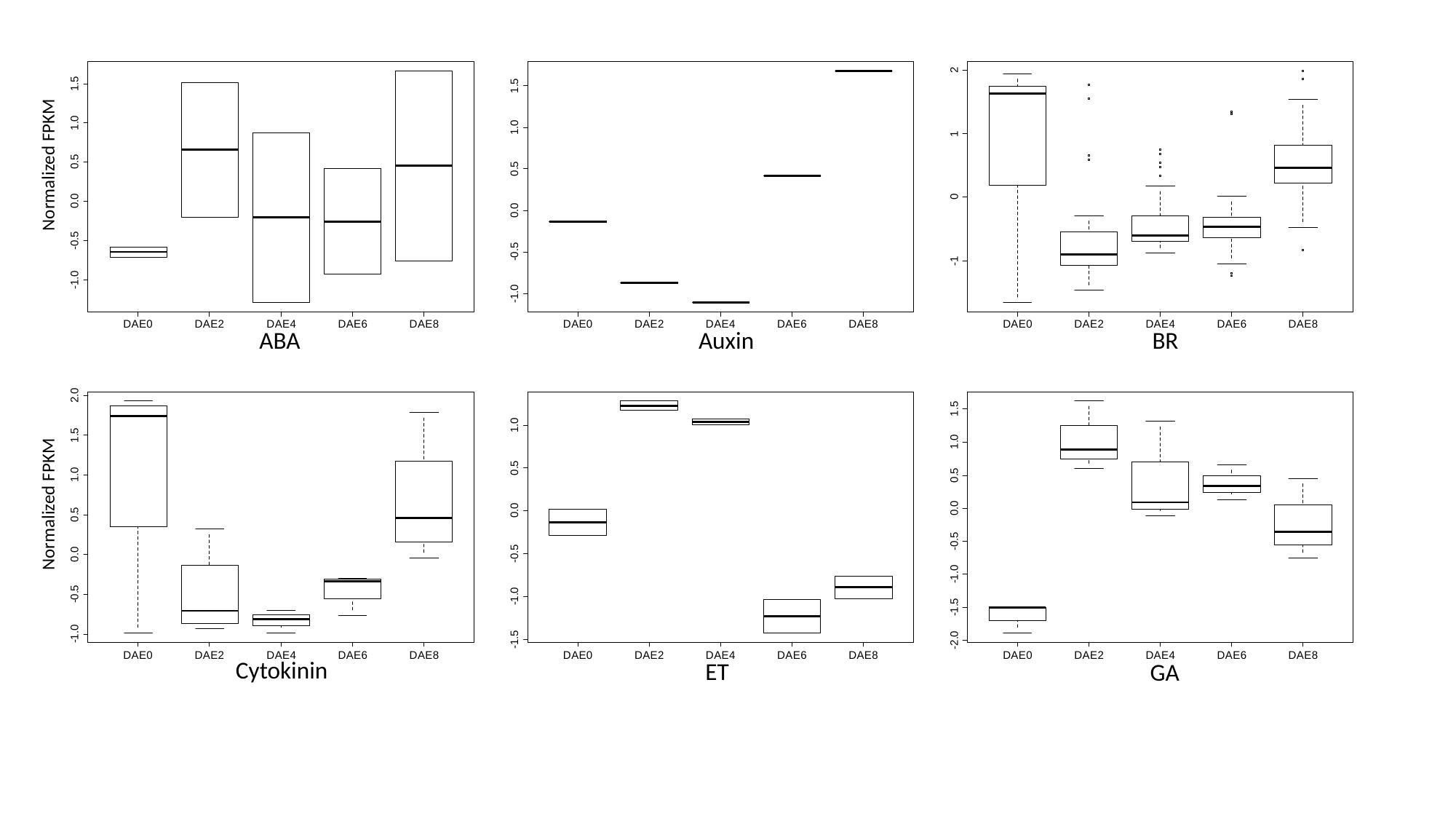

Normalized FPKM
ABA
BR
Auxin
Normalized FPKM
Cytokinin
ET
GA

Supplement: Supplementary file 8 — Figure S5. Boxplot of FPKM values for differentially expressed genes related to phytohormone receptors. The figure legends are similar to Additional file 7: Figure S3. (PPTX 45 kb) [file 12870_2019_1700_MOESM8_ESM.pptx]

## Slide 1
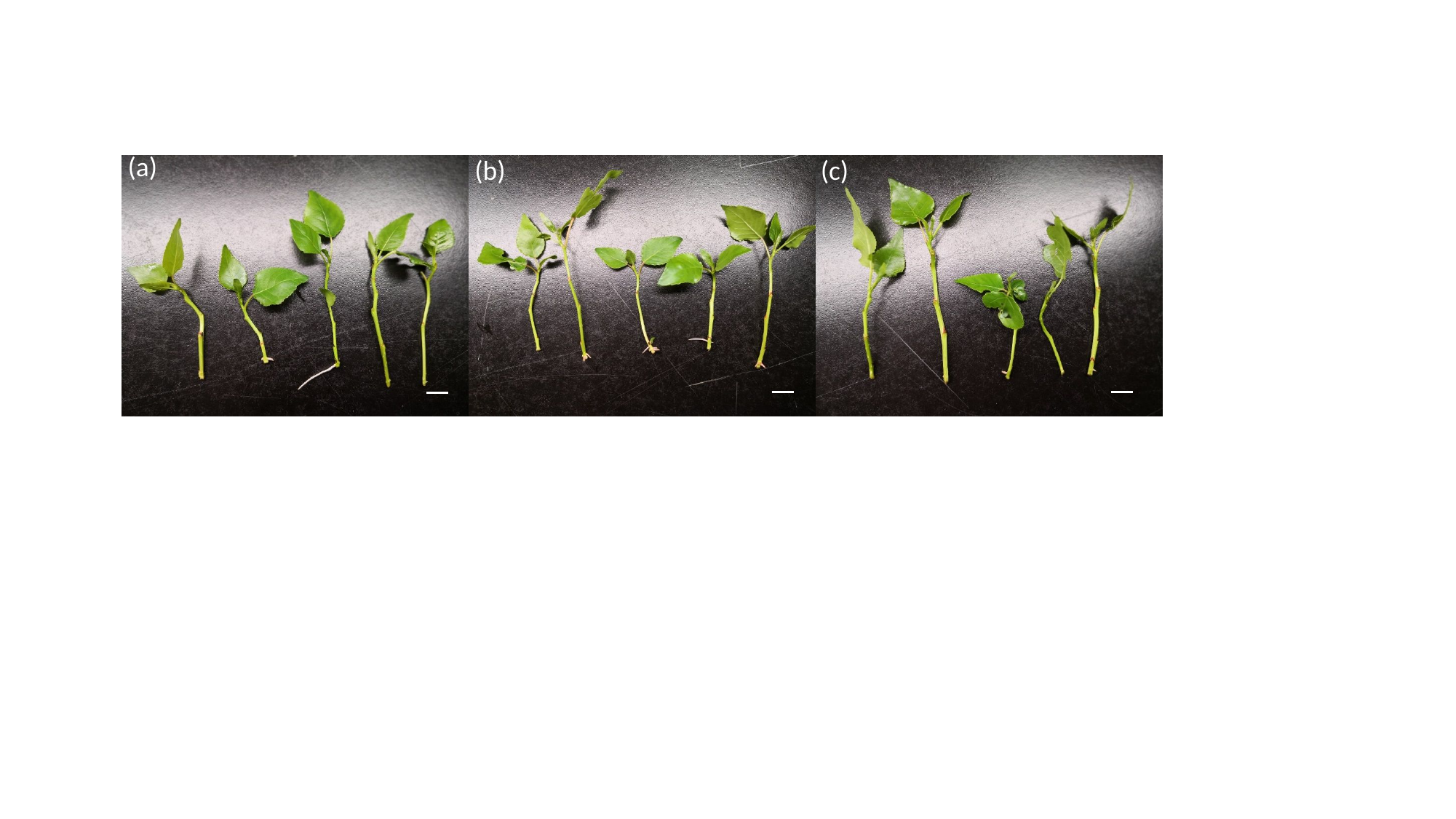

(a)
(b)
(c)

Supplement: Supplementary file 10 — Figure S6. Hydroponic culture of poplar cuttings with different concentrations of supplemented H2O2 at DAE11. Panels a, b, and c represent poplar cuttings grown in 0, 500, and 1000 μM H2O2, respectively. Bar = 1 cm. (PPTX 579 kb) [file 12870_2019_1700_MOESM10_ESM.pptx]
