# Supplementary material for: Comprehensive analysis of dynamic gene expression and investigation of the roles of hydrogen peroxide during adventitious rooting in poplar
Source: BMC Plant Biol. 2019 Mar 12;19:99. doi: 10.1186/s12870-019-1700-7 (PMC6416884; doi:10.1186/s12870-019-1700-7)
Supplement: Supplementary file 3 — Figure S2. Number of genes that are differentially expressed during poplar AR formation. (PPTX 9022 kb) [file 12870_2019_1700_MOESM3_ESM.pptx]

## Slide 1
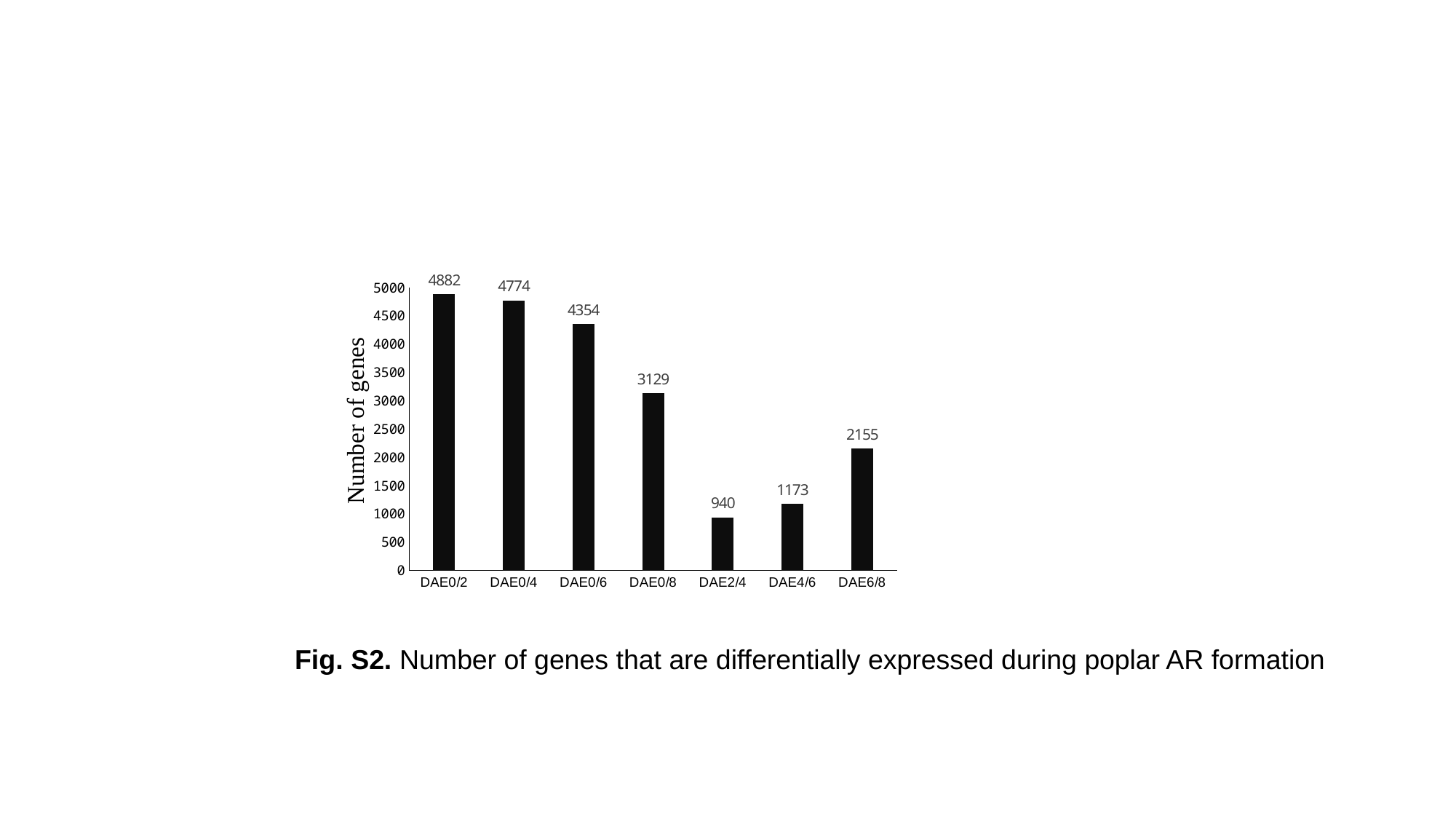

### Chart
| Category | |
|---|---|
| DAE0/2 | 4882.0 |
| DAE0/4 | 4774.0 |
| DAE0/6 | 4354.0 |
| DAE0/8 | 3129.0 |
| DAE2/4 | 940.0 |
| DAE4/6 | 1173.0 |
| DAE6/8 | 2155.0 |Number of genes
Fig. S2. Number of genes that are differentially expressed during poplar AR formation
